# Morphometric convergence among European sand gobies in freshwater (Gobiiformes: Gobionellidae)

**DOI:** 10.1002/ece3.5375

**Published:** 2019-06-20

**Authors:** Christine E. Thacker, Christos Gkenas

**Affiliations:** ^1^ Vertebrate Zoology, Collections and Research Santa Barbara Museum of Natural History Santa Barbara California; ^2^ Research and Collections, Section of Ichthyology Natural History Museum of Los Angeles County Los Angeles California; ^3^ Faculdade de Ciências MARE, Centro de Ciências do Mar e do Ambiente Universidade de Lisboa Lisboa Portugal; ^4^ Laboratory of Zoology Department of Biological Applications and Technology University of Ioannina Ioannina Greece

**Keywords:** convergence, Europe, freshwater, heterochrony, Mediterranean, phylogeny

## Abstract

The five genera of sand gobies inhabit the seas and freshwaters of Europe and western Asia and occupy habitats ranging from fully marine to exclusively freshwater. In this study, we use geometric morphometrics to quantify body shape among sand gobies, in order to investigate how shape has evolved and how it is related to habitat. We also compare body shape between preserved museum specimens and fresh specimens, to determine whether or not fixation and storage in ethanol introduce detectable bias. We confirm that the fixed specimens exhibit significant shape changes as compared to fresh specimens, and so, we perform the bulk of our analyses exclusively on fixed specimens. We find that *Economidichthys*, *Orsinigobius*, and *Pomatoschistus* occupy distinct regions of morphospace. *Knipowitschia* and *Ninnigobius* have intermediate forms that overlap with *Pomatoschistus* and *Orsinigobius*, but not *Economidichthys*. This pattern is also in rough accordance with their habitats: *Pomatoschistus* is fully marine, *Economidichthys* fully freshwater, and the others fresh with some brackish tolerance. We augment a recent phylogeny of sand gobies with data for *P. quagga* and interpret morphometric shape change on that tree. We then evaluate convergence in form among disparate lineages of freshwater species by constructing a phylomorphospace and applying pattern‐based (*convevol*) measures of convergence. We find that freshwater taxa occupy a mostly separate region of morphospace from marine taxa and exhibit significant convergence in form. Freshwater taxa are characterized by relatively larger heads and stockier bodies than their marine relatives, potentially due to a common pattern of heterochronic size reduction.

## 1 INTRODUCTION

The sand gobies are a group of five genera (*Pomatoschistus* Gill 1863, *Knipowitschia* Iljin 1927, *Economidichthys* Bianco, Bullock, Miller & Roubal 1987, *Ninnigobius* Whitley 1951, and *Orsinigobius* Gandolfi, Marconato & Torricelli 1986) that inhabit the marine, brackish, and fresh waters of Europe and western Asia. They are notable for the range of habitats they occupy, from the fully marine coastal *Pomatoschistus* species in the northeastern Atlantic and Mediterranean, to restricted range freshwater endemics in Italy, Croatia, Montenegro, Albania, and Greece (drainages of the Adriatic and Ionian seas). The group is monophyletic and part of the gobiiform family Gobionellidae (Thacker, 2013). Their relationships have usually been hypothesized with molecular data, because the species are all small and have few distinguishing morphological characters (Geiger et al., 2014; Huyse, Houdt, & Volckaert, 2004; Larmuseau, Huyse, Vancampenhout, Houdt, & Volckaert, 2010; Thacker, Gkenas, Triantafyllidis, Malavasi, & Leonardos, 2019; Vanhove et al., 2012). Those phylogenies have shown that the earliest‐diverging sand goby lineage is one of the freshwater genera, *Ninnigobius*, known from Adriatic drainages in Italy, Croatia, and Montenegro. Apart from *Ninnigobius*, the remainder of the sand gobies fall into two groups, the marine *Pomatoschistus* species and a clade containing the more brackish to freshwater *Knipowitschia*, *Orsinigobius*, and *Economidichthys*. In this study, we explore the significance of overall body shape in the sand gobies. We use geometric morphometrics to quantify body shape and then analyze the distribution of shape changes among species, between habitats, and in the context of their phylogeny. We also use pattern‐based tests of convergence to evaluate whether the freshwater species, which do not form a clade, exhibit significant convergence in body form. To provide an evolutionary framework for these tests, we generate a new sand goby phylogeny, adding to the dataset of Thacker et al. (2019) with newly generated sequence data for the enigmatic species *P. quagga*, derived from the study of Öztürk and Engin (2019).

**Figure 1:**
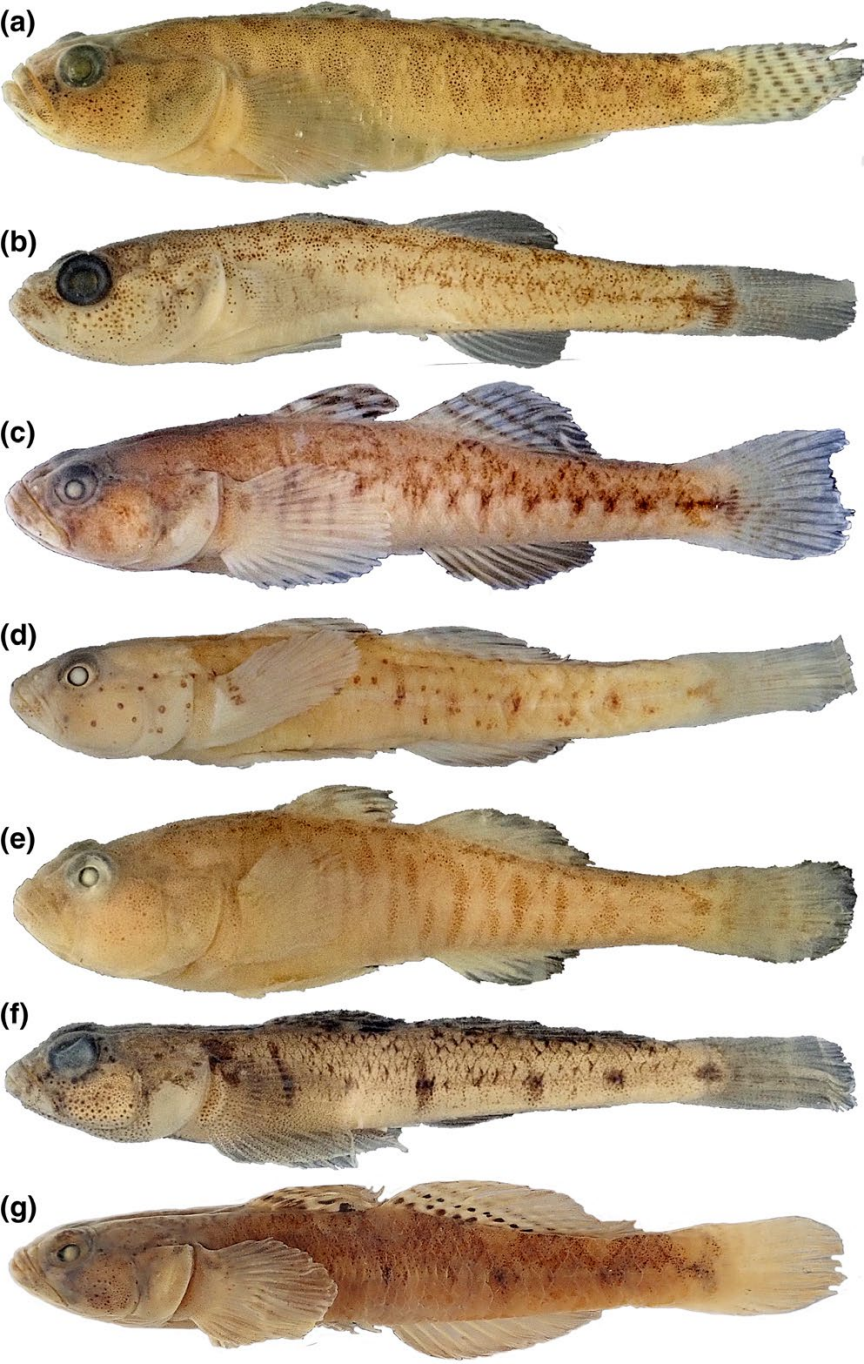
Representative sand goby individuals, from marine and freshwater. (a) *Economidichthys pygmaeus*, NMW86068, 32.3 mm; (b) *Knipowitschia milleri*, NMW86066 19.2 mm; (c) *Knipowitschia panizzae*, NMW29806 29.0 mm; (d) *Ninnigobius canestrinii*, NMW30618 32.9 mm; (e) *Orsinigobius punctatissimus*, NMW 87514 27.8 mm; (f) *Pomatoschistus marmoratus*, NMW87360 33.6 mm; (g) *Pomatoschistus pictus*, NMW28663 39.1 mm

Freshwater fish species face a different set of selection pressures from their habitats than marine fishes, given that their environment is usually smaller, more bounded, and potentially experiences more variable flow regimes. There are no overall generalizations concerning freshwater as opposed to marine fish body form, but among sand gobies there are some qualitative differences. Photographs of representative species from each sand goby genus are given in Figure 1. *Economidichthys* and *Orsinigobius* are deeper‐bodied, with proportionally larger heads, than their counterparts in *Pomatoschistus* and *Knipowitschia*. They are also generally smaller, attaining body sizes of 30–50 mm as adults, compared to 60–110 mm in most *Pomatoschistus* species, with *Knipowitschia* and *Ninnigobius* intermediate at 30–70 mm. Common morphological traits among freshwater species may relate directly to their ecology or may simply be a consequence of the smaller body size they all attain. Smaller fish species, particularly if they have achieved the size reduction by heterochrony, would be expected to have proportionally larger heads as well as reductions in characters such as scalation and head pores (Weitzman & Vari, 1988). Such reductions are seen in freshwater sand gobies (Miller, 1990). Miniature species may also exhibit unusual morphological novelties, such as the perianal organ found uniquely in *Economidichthys* species (Economidis & Miller, 1990; Hanken & Wake, 1993). The discrete characters associated with freshwater habitat preference in sand gobies are known, but the overall shape changes among the species have not been investigated. We seek to quantify the overall body shape among sand goby species and then interpret those patterns in the context of a phylogeny. This evolutionary perspective enables us to determine whether there are common aspects of shape change among freshwater sand goby species, and evaluate whether and to what degree those changes are concordant with the phylogeny, or convergent across lineages.

Morphological similarity among living organisms may result from a variety of causes, but inference of mechanism requires first quantifying the similarity, then ruling out the possibility that similar traits are simply due to inheritance from a common ancestor that also had those traits (Sanderson & Hufford, 1996). If similarity is confirmed, and phylogenetic commonality is ruled out, then it is reasonable to postulate other explanations for the pattern, including evolutionary convergence. Convergence may arise from a common selective pressure, functional constraint, ontogenetic constraint, some combination of these, or simply by chance (Losos, 2011). Notable examples of convergence include common locomotor morphologies among phylogenetically disparate but ecologically similar *Anolis* lizards (Losos, Jackman, Larson, Querioz, & Rodriguez‐Schettino, 1998), convergence of body shape and dentition morphology among feeding guilds in cichlids (Rüber & Adams, 2001), and convergence of head and jaw shape among herbivorous lizards (Stayton, 2006). In those cases, similar morphologies are exhibited in multiple, phylogenetically independent lineages (convergent pattern), and because the traits in question are easily relatable to function, a reasonable link between pattern and evolutionary process (parallel response to a common selective pressure) may be hypothesized. In other cases, convergence may be the result of a common evolutionary response induced by some separate selective change, such as patterns of trait simplification resulting from paedomorphic size reduction (Losos, 2011). Among sand gobies, the marine and freshwater species exhibit few consistent differences, but the overall body shapes do vary qualitatively. Using morphometric analysis coupled with phylogeny, we are able to evaluate the pattern of morphological evolution among these species and infer the evolutionary timing and cause.

Although many sand gobies are not uncommon where they occur, they are not often collected and so are generally rare in museum collections. We use ethanol‐fixed and preserved specimens from museum collections in this study and additionally compare the morphometric patterns to those derived from fresh specimens. We use a photographic dataset of six sand goby species (*Economidichthys pygmaeus* (Holly 1929), *Knipowitschia caucasica* (Berg 1916), *K. milleri* (Ahnelt & Bianco 1990), *K. panizzae* (Verga 1841), *Ninnigobius canestrinii* (Ninni 1883), and *Pomatoschistus marmoratus* (Risso, 1810)) from live collections made across the coastal localities and fresh waters of Greece and the Adriatic (Venice Lagoon). We compare those individuals to fixed specimens of the same species to determine whether or not fixation introduces a bias in analysis of landmark data, and if so, whether or not that bias is consistent and what form it takes. Previous works comparing geometric morphometric analysis of fixed and fresh individuals from both marine and freshwater fish species determined that fixation did introduce a significant bias, specifically manifested as overall shrinkage and decrease in eye diameter (Berbel‐Filho, Jacobina, & Martinez, 2013; Martinez, Berbel‐Filho, & Jacobina, 2013).

To evaluate evolutionary patterns in body form, we use a more comprehensive dataset of fourteen sand goby species, the data for which are all derived from fixed specimens. These species include the most common and widespread sand goby species and span all the clades within the sand goby phylogeny of Thacker et al. (2019). Due to the rarity of sand gobies in museum collections, and the need for intact adult specimens for morphometric analysis, we were unable to assemble data for the rarer species. We examined between three and 26 individuals for each species used, except for *Pomatoschistus lozanoi* (de Buen 1923) for which only one appropriate specimen was available. We then combine those data with a phylogenetic hypothesis, augmented from a previous sand goby phylogeny (Thacker et al., 2019). We evaluate the phylogenetic significance of shape change and investigate the degree to which freshwater species have converged.

## 2 MATERIALS AND METHODS

### 2.1 Landmark acquisition and analysis

We examined a total of 245 sand goby specimens from 14 species, 127 from museum collections (ethanol fixed and preserved) and 118 freshly caught, photographed shortly after death. Fixed specimens were examined at the Naturhistorisches Museum, Vienna, and the Natural History Museum, London, and additional specimen photographs were provided by the Muséum National d'Histoire Naturelle, Paris. The specimens were collected between 1874 and 1995, with most dating back to before the early 1900s, at localities spanning the northeastern Atlantic, Mediterranean, and Adriatic Seas and associated freshwaters. We selected specimens that were adult, undamaged, and as unbent as possible and photographed all specimens in left lateral view with an Olympus Tough TG‐5 camera, using a copystand and the automatic z‐stacking option. Although many of the specimens were in good condition, considering their age, we were limited in both the numbers available and in selecting specimens suitable for photography and morphometric analysis. Fresh specimens were collected, either using hand nets or electrofishing gear, from sixteen localities on the Aegean and Ionian coasts of Greece and the Adriatic (Venice Lagoon). Individual fish were euthanized by immersion in tricaine methanesulfonate (MS‐222) and photographed before preservation. Species and numbers of individuals examined are given in Table 1.

**Table 1:** Species of sand gobies used in this study, with counts of formalin‐fixed and fresh specimens used in geometric morphometric analyses, habitat (FW = freshwater; BR = brackish; MA = marine), and for the freshwater/brackish species, whether or not the species inhabits drainages of the Adriatic or Ionian seas (waterways in Italy, Slovenia, Croatia, Bosnia, and Herzegovina, Montenegro, Albania, and western Greece)

Species	Fixed	Fresh	Habitat	Adriatic/Ionian
*Economidichthys pygmaeus*	12	50	FW	Yes
*Knipowitschia caucasica*	3	19	FW BR MA	Yes
*Knipowitschia milleri*	9	33	FW	Yes
*Knipowitschia panizzae*	13	6	FW BR	Yes
*Ninnigobius canestrinii*	10	5	FW BR	Yes
*Orsinigobius punctatissimus*	6	—	FW	Yes
*Pomatoschistus flavescens*	5	—	MA	—
*Pomatoschistus lozanoi*	1	—	MA	—
*Pomatoschistus marmoratus*	26	5	MA	—
*Pomatoschistus microps*	7	—	MA	—
*Pomatoschistus minutus*	12	—	MA	—
*Pomatoschistus norvegicus*	3	—	MA	—
*Pomatoschistus pictus*	17	—	MA	—
*Pomatoschistus quagga*	3	—	MA	—

**Figure 2:**
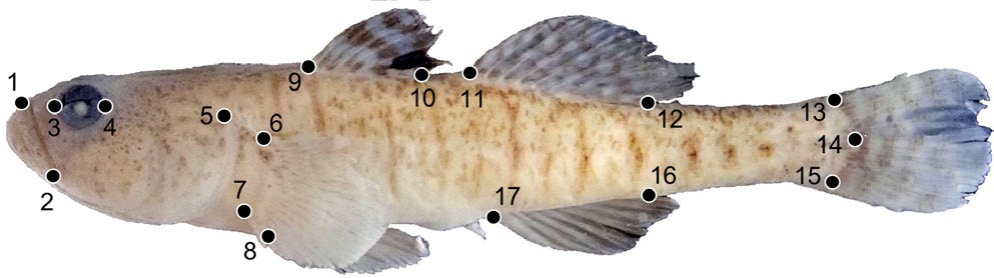
Landmarks digitized in this study, superimposed on *Knipowitschia caucasica* NMW91653‐3, 32.3 mm

We digitized 17 landmarks for each individual, using ImageJ version 1.52 a (Schneider, Rasband, & Eliceiri, 2012), as shown in Figure 2. This suite of external landmarks describes the overall body shape, the fin positioning, and the locations and size of the mouth and eyes. They have been used previously to quantify shape variation in gobioid species (Thacker, 2014, 2017) and are reliably assignable to both preserved and fresh specimens. All landmarks were assigned in all specimens examined. We then forwarded the landmark coordinates to MorphoJ version 1.05d (Klingenberg, 2011), performed a Procrustes fit, generated a covariance matrix, and used that matrix as input for principal components analysis (PCA). We first analyzed paired landmark data for six species for which we had both preserved and fresh specimen data: *Economidichthys pygmaeus*, *Knipowitschia caucasica*, *K. milleri*, *K. panizzae*, *Ninnigobius canestrinii*, and *Pomatoschistus marmoratus*. For those data, we used PCA to evaluate whether or not individuals of the same species could be separated on the basis of preservation. We additionally used MorphoJ to perform a discriminant function analysis (DFA), grouping individuals by preservation technique, to compare morphometric distance between fixed and fresh specimens and assess its significance. For both the PCA and the DFA, we analyzed each of the six species separately and also all of the species together. Finally, we imported the landmark data into R (version 3.5.0) and used *geomorph* (version 3.0.7; Adams & Otarola‐Castillo, 2013), to perform Procrustes ANOVA (ANOVA of Procrustes coordinates) and further test for significant shape divergence between the fixed and fresh individuals.

We found that preservation technique introduced a significant bias to the shape data, so we proceeded with our morphometric and comparative analyses using only fixed museum specimens. Our dataset included 14 sand goby species: *Economidichthys pygmaeus*,* Knipowitschia caucasica*,* K. panizzae*,* Ninnigobius canestrinii*,* Orsinigobius punctatissimus* (Canestrini 1864),* Pomatoschistus flavescens* (Fabricius 1779),* P. lozanoi*,* P. marmoratus*,* P. microps* (Krøyer 1838),* P. minutus* (Pallas 1770),* P. norvegicus* (Colett 1902),* P. pictus* (Malm 1865), and *P. quagga* (Heckel 1839); species and numbers of individuals examined are given in Table 1, and museum catalog numbers for material examined are given in the Appendix (Table A1). For these data, we digitized landmarks and again used MorphoJ to generate a PCA, as well as a canonical variates analysis (CVA) grouping the individuals by genus and by habitat preference (fresh, brackish, or marine). We performed Procrustes ANOVA (not corrected for phylogenetic relationships) with *geomorph*, regressing shape change against species, genus, and habitat.

### 2.2 Phylogenetic comparative analyses and tests of convergence

Sand goby species are all part of the same clade, so comparisons among species are not phylogenetically independent. To correct for this bias, we used phylogenetic comparative methods to evaluate shape change and test for convergence in morphology by combining morphometric landmark data with a phylogeny of species. We expanded upon the calibrated phylogeny from Thacker et al. (2019) for sand gobies, adding newly published sequence for *Pomatoschistus quagga* to that mitochondrial COI dataset (Öztürk & Engin, 2019; GenBank numbers MK302484‐8, all GenBank accession numbers for specimens used in this analysis are given in the Appendix (Table A2). We assembled the matrix using Geneious (Biomatters, Ltd.) version 10.2.6 and performed a Bayesian search as originally described: 10 × 10^7^ generations using a GTR + I + G substitution model, run with four simultaneous chains, sampling every 1,000 replications and discarding the first 10% of trees as burn‐in. We constructed a 50% majority‐rule consensus tree, trimmed the hypothesis to two exemplars of each species (three for widespread *P. marmoratus*), and then calibrated the phylogeny using BEAST 1.7.5 (Drummond, Suchard, Xie, & Rambaut, 2012). We used three fossil calibrations from Schwarzhans et al. (2017) for the origins of *Economidichthys* (15.0 Mya), *Pomatoschistus* (excluding *P. quagga*, in accordance with the Bayesian results; 16.0 Mya), and *Knipowitschia* (13.0 Mya), applied as exponential priors, and a legacy calibration from the phylogeny of Thacker (2015) for the origin of Gobionellidae at 48.7 Mya, assigned as a normal prior. We then trimmed the phylogeny to single exemplars of species for which we had shape data using Mesquite version 3.1.0 (Maddison & Maddison, 2018) for use in the comparative analyses. All trees were visualized with FigTree 1.4.2 (Rambaut, 1999).

We combined the calibrated phylogeny with the morphometric data, tested for phylogenetic signal, and then performed phylogenetic MANOVA on the scores for the first six uncorrected PC axes (accounting for 82% of the variance), averaged by species, using the R packages *geomorph* (version 3.0.7; Adams & Otarola‐Castillo, 2013), and *geiger* (version 2.0.6; Harmon, Weir, Brock, Glor, & Challenger, 2012). The phylogenetic MANOVA (aov.phylo) in *geiger* is performed by first computing the *F*‐statistic as in a normal MANOVA. Then, to evaluate significance, *geiger* simulates a null distribution of dependent variables (in this case, PC scores) on the phylogeny using a Brownian motion model, determines the *F*‐statistic for each replicate, and compares that distribution to the value obtained for the observed data (Garland, Dickerman, Janis, & Jones, 1993). Finally, we used *phytools* (version 0.6‐44; Revell, 2012) to generate a phylomorphospace (phylogeny superimposed on a plot of PC1 vs. PC2; Sidlauskas, 2008).

To test for convergent evolution of shape among the freshwater species, we used the four pattern‐based measures of convergence implemented in *convevol* (version 1.3; Stayton, 2015). These four measures (C1–C4) evaluate the pattern of convergence among taxa in a morphospace. C1 describes the amount of morphospace difference attributable to convergence, C2 is the absolute magnitude of change for convergent lineages in morphospace, and C3 and C4 are relative measures: C3 = C2/total lineage change and C4 = C2/total clade change. In all cases, significance is assessed by comparing the observed pattern to 500 generations of a Brownian motion simulation of trait (habitat) data across the phylogeny.

For habitat and range information, we scored each species using information from field guides (Kottelat & Freyhof, 2007; Louisy, 2015; Miller, 2004; Miller & Loates, 1997) and FishBase (Froese & Pauly, 2018). We performed the habitat coding in two ways. First, we coded habitat as a multistate character, encompassing exclusively freshwater, freshwater/brackish, freshwater/brackish/marine, brackish/marine, or exclusively marine. This coding is more accurate, but introduces an artifact in that overlapping conditions (for instance, freshwater vs. freshwater/brackish) are treated as independent states. We used this multistate coding for the Procrustes ANOVA and phylogenetic MANOVA. We also performed the Procrustes ANOVA, phylogenetic MANOVA, and convergence tests with a binary coding scheme, using marine for the exclusively marine *Pomatoschistus* species, and freshwater for the other species, all of which inhabit fresh or brackish water. This binary scheme should more accurately test our primary question, which is whether or not species that are exclusively or mostly known from freshwater exhibit any common morphometric pattern.

## 3 RESULTS

### 3.1 Morphometrics of fixed and fresh specimens

**Figure 3:**
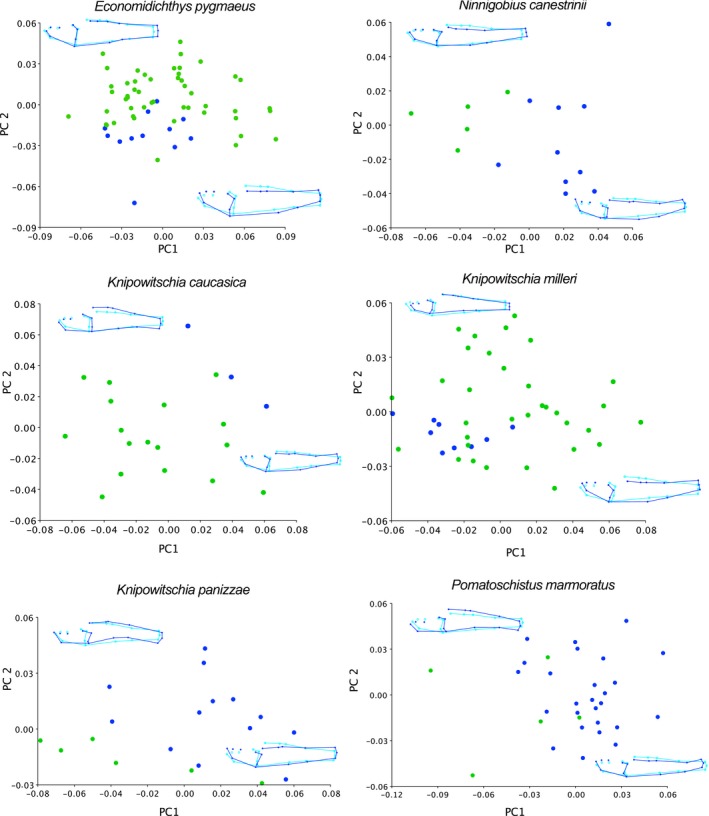
Morphometric PCA plots for fresh versus fixed specimens of six sand goby species: *Economidichthys pygmaeus*, *Ninnigobius canestrinii*, *Knipowitschia caucasica*, *K. milleri*, *K. panizzae*, and *Pomatoschistus marmoratus*. In all graphs, fresh specimens are indicated with green dots, fixed specimens in blue

The morphometric PCA plots for fresh versus fixed specimens are given in Figure 3. In most of the comparisons (except *Economidichthys pygmaeus* and *Knipowitschia milleri*), the fixed and fresh specimens are nearly or completely separated in plots of PC1 versus PC2. For *E. pygmaeus*, *K. milleri*, *K. caucasica*, and *Pomatoschistus marmoratus*, the first two PC axes explained 50%–58% of the total variance; for *K. panizzae* and *Ninnigobius canestrinii*, that total was 67%. Because each of these plots represents a separate PCA, the axes are not equivalent among species, however, when all the species are analyzed together and thus compared to a common reference configuration, the results are nearly identical. Results from the separate PCAs are shown in Figure 3 because it is much easier to interpret the graphical patterns if the separate species are not overlain. In both the combined and separate PCAs, the wireframes displaying shape change on PC1 and PC2 all describe changes in body depth and tail length and curvature, known artifacts of fixation on body shape (Berbel‐Filho et al., 2013; Martinez et al., 2013). DFAs comparing fixed and fresh individuals, with species analyzed separately or together, were significantly different based on 1,000 permutations of the T‐square statistic (*p* < 0.0001) for every species except for *P*. *marmoratus* (*p* = 0.098). Procrustes ANOVAs of shape change between fixed and fresh specimens of each species were nearly significant (*p* = 0.056, *N. canestrinii*), significant (*p* = 0.01, *K. panizzae*) or highly significant (*p* = 0.001–0.007, all other species, consistent with greater statistical power associated with larger sample sizes).

To further evaluate the effect of fixation on shape change, we examined the Procrustes distance between fixed and fresh specimens for each species. We first compared the Procrustes distances obtained from the separate and combined DFAs, and for each species, they were identical to the third decimal place. Distances between fixed and fresh specimens ranged from 0.034 (*Economidichthys pygmaeus*) to 0.067 (*Knipowitschia caucasica*), with a mean of 0.052. These estimates are comparable to the distances among fixed species, which range from 0.026–0.132, with a mean of 0.065. Given these artifacts, we did not combine the fixed and fresh specimens into a single dataset for further analysis. Instead, we proceeded with exclusively fixed museum specimens.

### 3.2 Sand goby phylogeny

**Figure 4:**
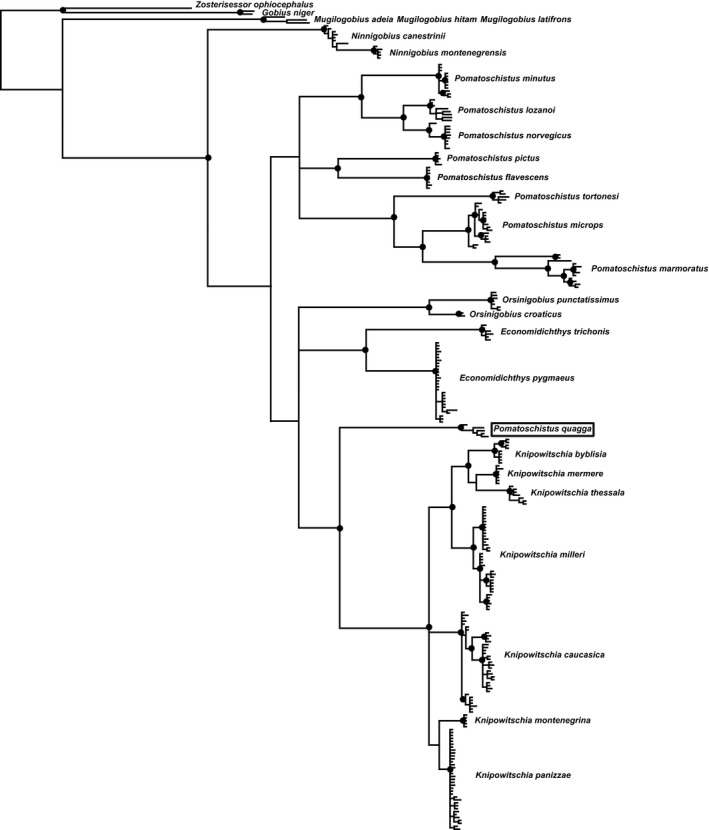
Phylogeny of sand gobies, derived from Bayesian analysis of partial mitochondrial COI sequence for 270 individuals of sand gobies and six gobioid outgroup sequences. Black circles at nodes indicate 95%–100% posterior probability. This hypothesis supports the monophyly of each sand goby genus, with the exception of *Pomatoschistus*. The unusual *P. quagga* (indicated with black box) is placed as sister to *Knipowitschia*, outside the remainder of *Pomatoschistus*

The phylogeny of sand gobies is given in Figure 4. This hypothesis is based on the same data as Thacker et al. (2019), with the addition of *Pomatoschistus quagga*. The hypothesis is nearly identical to that of Thacker et al. (2019), except that here we recover a monophyletic *Pomatoschistus*, to the exclusion of *P. quagga*. *Pomatoschistus quagga*, an unusual *Pomatoschistus* species that inhabits the western Mediterranean and Adriatic seas, is resolved as sister to the genus *Knipowitschia* and within a clade that also includes *Economidichthys* and *Orsinigobius* (resolved here as sequential sister taxa to *Knipowitschia*, rather than as a distinct clade). This placement of *P. quagga* has been obtained in previous studies, using different molecular markers (Huyse et al., 2004; Vanhove et al., 2012). *Pomatoschistus* is a fully marine genus, unlike the freshwater/brackish preferring *Knipowitschia*, *Economidichthys* and *Orsinigobius*. Due to this placement of *P. quagga* outside the remainder of *Pomatoschistus*, we performed the morphometric and phylogenetic comparative analyses with *P. quagga* designated as a distinct lineage (genus). Other relationships among species and genera are as inferred by Thacker et al. (2019), including monophyly of *Ninnigobius*, *Orsinigobius*, *Economidichthys*, and *Knipowitschia*; placement of *Ninnigobius* outside the remainder of sand gobies, and a grouping of *Economidichthys*, *Orsinigobius*, and *Knipowitschia*.

**Figure 5:**
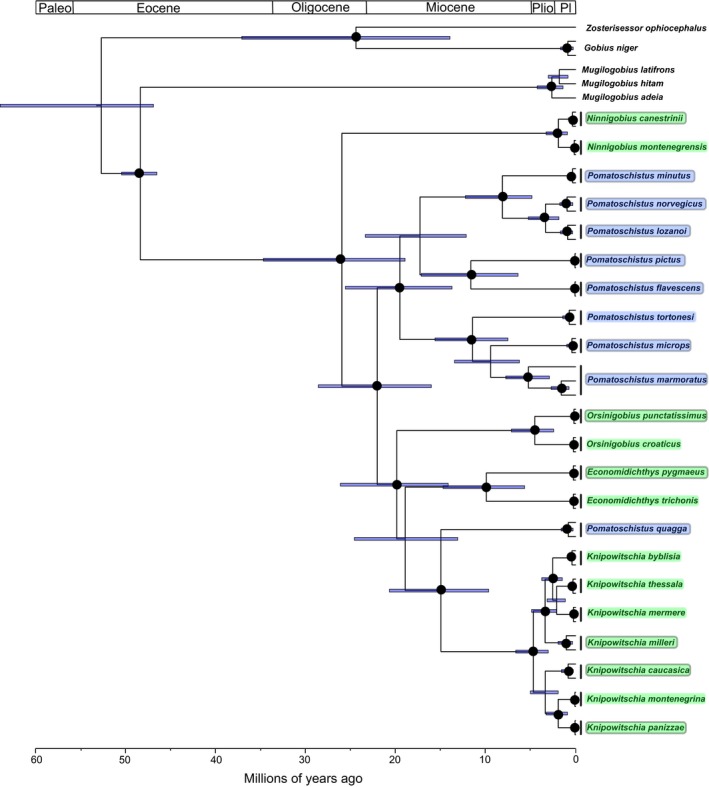
Calibrated sand goby phylogeny, trimmed from that shown in Figure 4 to a total of 51 individuals, 45 sand gobies plus six outgroup sequences, and calibrated with three fossil and one legacy calibrations. Black circles at nodes indicate 95%–100% posterior probability, and error bars indicate 95% highest posterior density of calibration estimates. Among the sand gobies, blue shading on species names indicates marine habitat, green shading indicates freshwater tolerance (FW or FW/BR habitat preference), and species examined for morphometric shape data are circled. Freshwater ecology is not confined to a single clade; it is present in *Ninnigobius*, *Economidichthys*, *Orsinigobius*, and *Knipowitschia*

The calibrated phylogenetic hypothesis, based on representative individuals for each species, is given in Figure 5. The age estimates for nodes are very close (within 95% confidence intervals) to those inferred in Thacker et al. (2019), except for the crown ages of the genera *Knipowitschia*, which is here estimated at 4.7 Mya (95% confidence interval 3.1–6.8 Mya) rather than 13.3 Mya (13.0–14.4 Mya), and *Economidichthys*, here estimated at 10.0 Mya (5.7–14.8 Mya), rather than 15.5 Mya (15.0–17.1). Also indicated on the calibrated phylogeny are the habitat preferences for each species, as assigned in the binary coding for the comparative phylogenetic analyses (marine vs. freshwater).

### 3.3 Sand goby morphometrics

**Figure 6:**
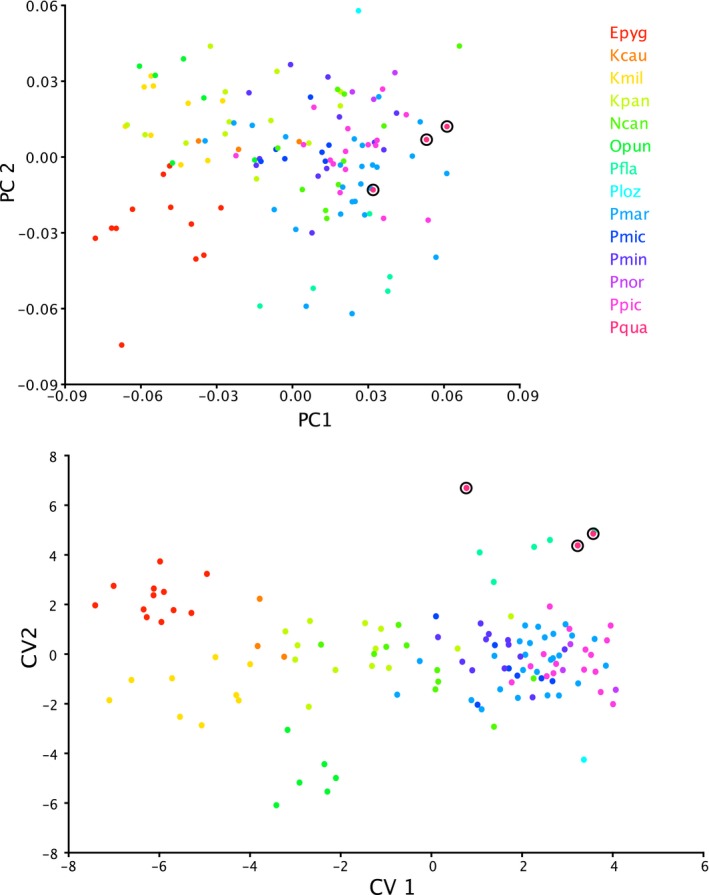
Morphometric PCA (top) and CVA (bottom) plots for fixed specimens only. Color legend applies to both graphs, freshwater tolerant species are shown in shades of red, orange, yellow, and green, while marine species are shown in shades of blue, purple, and pink. Individuals of *P. quagga* are circled. Epyg = *Economidichthys pygmaeus*; Kcau = *Knipowitschia caucasica*; Kpan = *K. panizzae*; Kmil = *K. milleri*; Ncan = *Ninnigobius canestrinii*; Opun = *Orsinigobius punctatissimus*; Pfla = *Pomatoschistus flavescens*; Pmin = *P. minutus*; Pnor = *P. norvegicus*; Ppic = *P. pictus*; Pmic = *P. microps*; Pmar = *P. marmoratus*; Ploz = *P. lozanoi*; Pqua = *P. quagga*

The dataset of fixed museum specimens included 14 species of sand gobies and encompassed most of the widespread common species found in the seas and freshwaters of Europe. Plots for PC1 versus PC2 of morphometric data are given in Figure 6. The first two PC axes account for 50% of the total variance (35% and 15%, respectively). The shape change on PC1 is dramatic, describing a transformation between a slender body and relatively small head to a chunkier body and relatively much larger head. The change on PC2 is more slight and is largely concentrated in an extension of the caudal region, resulting in a more elongate form. For both the PCA and the CVA, the first axis describes most of the variation among species, with further separation among similar freshwater species apparent on the second axis. Marine species (*Pomatoschistus*) are not as distinct from one another in either the PCA or the CVA, except for *P. quagga*, which is within the *Pomatoschistus* morphospace range on both PC axes but separated from the other species on CV2. CVAs by genus yielded highly significant (*p* < 0.0001) distinctions among all of the traditional genera (*Economidichthys*, *Knipowitschia*, *Ninnigobius*, *Orsinigobius*, *Pomatoschistus*) and were significant at *p* < 0.001 for comparisons involving *P. quagga*. CVA by habitat preference also yielded significant (most comparisons *p* < 0.0001, comparisons among FW, FW/BR and FW/BR/MA significant at *p* < 0.001) divergence in body shape. Procrustes ANOVAs of shape for all individuals yielded highly significant (*p* < 0.001) differences among species, among genera, and among habitats; habitat differences were significant with both the multistate and binary habitat coding.

### 3.4 Phylogenetic comparative analyses and tests of convergence

We detected significant phylogenetic signal among the shape data (*p* = 0.044) based on the Kmult test. The phylogenetic MANOVA of PC scores was nearly significant for genus (*p* = 0.059), but not for habitat (*p* = 0.431 for multistate habitat coding, *p* = 0.216 for binary habitat coding). Tests of convergence among the freshwater taxa were highly significant. The C1 value, indicating percentage of morphometric distance accounted for by convergence, was 0.402 (40.2%). The magnitude of convergent change (C2) was 0.012 (highly significant), as were the relative measures C3 and C4. In contrast, applying the convergence tests with marine species (*Pomatoschistus)* designated as potentially convergent yields insignificant results. *Convevol* results are given in Table 2.

**Table 2:** Results from *convevol* analysis

Species group	C1	C2	C3	C4
Freshwater	0.402*	0.012*	0.277*	0.062*
Marine	−0.170	−0.002	−0.082	−0.009

Note

Freshwater = *Economidichthys pygmaeus*, *Knipowitschia caucasica*, *K. milleri*, *K. panizzae*, *Ninnigobius canestrinii*, and *Orsinigobius punctatissimus*. Marine = all species of *Pomatoschistus* (including *P. quagga*).

* Indicate significance at the *p* < 0.001 level.

**Figure 7:**
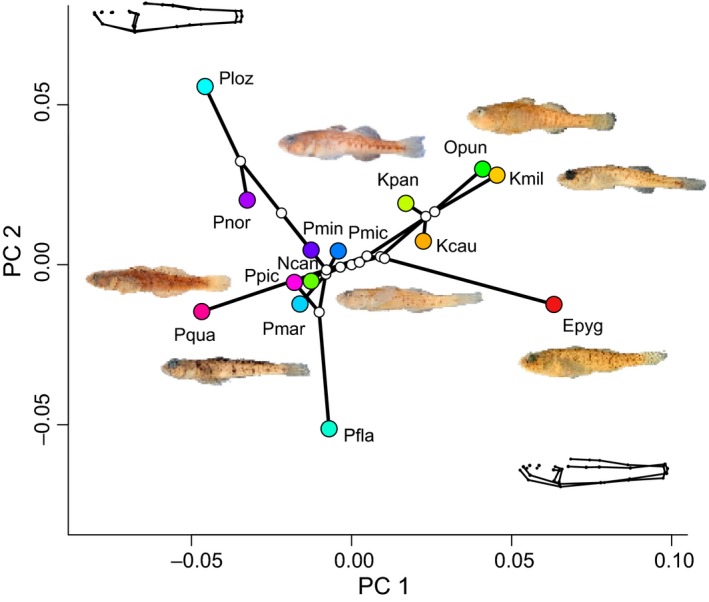
Tree of the 14 species used in phylogenetic comparative analyses, plotted as a phylomorphospace (phylogeny superimposed on a plot of PC1 vs. PC2). Wireframes show the type of change on each axis, overall broadening of the body and head for PC1, and elongation of the body for PC2. Images of fishes are those depicted in Figure 1. Clockwise from top center they are *Knipowitschia panizzae*, *Orsinigobius punctatissimus*, *K. milleri*, *Economidichthys pygmaeus*, *Ninnigobius canestrinii*, *Pomatoschistus pictus*, and *P. marmoratus*. Abbreviations on terminal taxa are Epyg = *E. pygmaeus*; Kcau = *K. caucasica*; Kpan = *K. panizzae*; Kmil = *K. milleri*; Ncan = *N. canestrinii*; Opun = *O. punctatissimus*; Pfla = *P. flavescens*; Pmin = *P. minutus*; Pnor = *P. norvegicus*; Ppic = *P. pictus*; Pmic = *P. microps*; Pmar = *P. marmoratus*; Ploz = *P. lozanoi*; Pqua = *P. quagga*. Colored circles for terminal taxa correspond to the color scheme used in Figure 6: Freshwater taxa are indicated with shades of red, orange, yellow, and green; marine taxa are denoted with shades of purple, pink, and blue

The phylomorphospace of sand goby species is shown in Figure 7. Generally, the freshwater genera (*Knipowitschia*, *Orsinigobius*, and *Economidichthys*) occupy the right side of the phylomorphospace, with marine *Pomatoschistus* species arrayed on the left side. The exception is *Ninnigobius*, which is placed slightly inside the *Pomatoschistus* morphospace, nearer to *P. pictus* and *P. marmoratus* than to the other freshwater genera. The separation between these groups is most strongly loaded on PC1, with PC2 representing elongation in the caudal region and providing differentiation among species within the two ecological types.

## 4 DISCUSSION

### 4.1 Morphometric distinctions between fixed and fresh specimens

Sand gobies are small and elongate and so may be particularly affected by the shrinkage and deformation induced by fixation. Our comparisons of fresh and fixed individuals of six sand goby species confirm this pattern. Four out of the six species examined exhibit complete or near‐complete separation between fixed and fresh specimens on plots of PC1 versus PC2 (Figure 3), both Procrustes ANOVAs and DFAs yielded significant or near‐significant differences in every comparison, and Procrustes distances among fixed and fresh specimens of the same species were comparable to the distances between fixed species. Fixation in ethanol has been shown to yield a small but significant reduction in body length in both marine and freshwater fishes, with most effects seen in the first 20 days after preservation (Buchheister & Wilson, 2005; Fey & Hare, 2005; König & Borcherding, 2012; Thorstad et al., 2007). Differences in overall shape, quantified with geometric morphometrics, were also confirmed for formalin fixation in two marine species (Martinez et al. (2013) and for ethanol fixation in a freshwater species (Berbel‐Filho et al., 2013). Both of those studies found that significant errors were introduced into the shape data by fixation, most prominently manifested as a shrinkage in the eyes and overall body depth. In our more terete, elongate specimens, we confirm reductions in body depth and overall length for fixed specimens, as well as deformation of the caudal region. There are slight differences in relative eye placement between the fixed and fresh specimens, but we did not detect notable shrinkage of the eyes. Interestingly, the changes in shape due to fixation were not consistent across species. Four of the six fixed/fresh specimen comparisons displayed an increase in body flexion and a decrease in overall length in the fixed individuals (*Knipowitschia caucasica*, *K. panizzae*, *Ninnigobius canestrinii*, and *Pomatoschistus marmoratus*; Figure 3), but the other two (*Economidichthys pygmaeus* and *K. milleri*) had fixed and fresh individuals overlapping in the plots, without clear distinction between the groups. This may be due to the fact that PC eigenvectors, in contrast to simple linear measurements, describe complex overall shape changes and may vary in which axis of change they detect as the primary one. However, in all comparisons, the DFA's and Procrustes ANOVAs showed significant or near‐significant differences between fixed and fresh specimens. To avoid any confounding effects of including both fixed and fresh specimens, and because the majority of specimens available for morphometric analyses were fixed museum specimens, we proceeded with fixed specimens only.

### 4.2 Sand goby phylogeny and timing

Our phylogeny combines the matrix used in Thacker et al. (2019), derived from the earlier studies of Costa et al. (2012), Geiger et al. (2014), Knebelsberger and Thiel (2014), and Landi et al. (2014), with new data for *Pomatoschistus quagga* from the work of Öztürk and Engin (2019). The primary novel result of this combined hypothesis is the recovery of a monophyletic *Pomatoschistus*, to the exclusion of *P. quagga*. These changes also affect the divergence time estimates of Thacker et al. (2019), most notably placing the crown age of *Economidichthys* later (10.0 Mya as opposed to 15.5 Mya), and the crown age of *Knipowitschia* much later (4.7 Mya as opposed to 13.3 Mya) than the previous estimates. Other than that, all divergence times are within the error estimates of the previous hypothesis, and the overall biogeographic interpretations are unchanged. Our hypothesis indicates that sand gobies arose in the late Oligocene (26.1 Mya), followed by a radiation of genera (and the *P. quagga* lineage) up to the mid‐Miocene (the split between *Knipowitschia* and *P. quagga* is the latest intergeneric split at 15.0 Mya). We estimate the origins (stem ages) of *Pomatoschistus* (excluding *P. quagga*) at 22.1 Mya, *Knipowitschia* at 15.0 Mya, *Economidichthys* at 19.0 Mya, *Orsinigobius* at 19.9 Mya, and *Ninnigobius* at 26.1 Mya. *Pomatoschistus* is the oldest generic radiation, with a crown age of 19.6 Mya (early Miocene), followed by *Economidichthys* (10.0 Mya, late Miocene). *Orsinigobius* (4.5 Mya) and *Knipowitschia* (4.7 Mya, with the *K. milleri* and *K. caucasica* clades each originating at 3.4 Mya) diversified in the Pliocene, followed by *Ninnigobius*, the earliest‐diverging genus but the one with the youngest crown age, estimated at 1.9 Mya in the Pleistocene. These results are consistent with the fossil records of *Pomatoschistus*, *Knipowitschia*, and *Economidichthys* discussed in Schwarzhans et al. (2017).

The earlier (Miocene) dates for diversification of genera and of species within *Pomatoschistus*, correlate roughly with the closure of the Tethys seaway, isolating the Mediterranean and North Atlantic from the tropical Indian Ocean. *Economidichthys* is the earliest freshwater clade to diversify, also during the Miocene, and likely linked to the emergence of the Greek peninsula as sea levels fell (Rögl, 1999a, 1999b). The later pulse of diversification among sand gobies occurred in the late Miocene to Pliocene and was centered in the Adriatic, the center of diversity for the group, particularly for the freshwater species. The inferred dates for the radiations of *Knipowitschia* (4.7 Mya, 95% confidence interval 3.05–6.67 Mya) and *Orsinigobius* (4.5 Mya, 95% confidence interval 2.44–7.16 Mya) correspond roughly to the Messinian salinity crisis (MSC), a set of drastic environmental changes that proceeded from the near‐total desiccation of the Mediterranean basin, due to the closure of the strait of Gibraltar, from approximately 5.96 to 5.33 Mya (Marzocchi, Flecker, Baak, Lunt, & Krijgsman, 2016; Rögl, 1998). The termination of the MSC occurred when the strait of Gibraltar reopened, resulting in the reflooding of the Mediterranean (the Zanclean flood). Near the end of the MSC, increases in rainfall delivered more freshwater to the rivers and brackish basins, including the inland Paratethys sea to the north and east (a Tethyan relic that was isolated from the Mediterranean during the Oligocene by the uplift of the Alps, Carpathians, and Dinarides mountains). The Paratethys encompassed the current basins of the Black and Caspian seas and extended westward to include the Dacic Basin (roughly modern‐day Romania) and the Pannonic Basin (roughly modern‐day Hungary and Slovenia). Over a short interval, from 5.43 to 5.33 Mya, fresh to brackish water incursions from the Paratethys flowed into the basins of the Adriatic and Aegean seas. These incursions resulted in the Lago Mare phase, with brackish water filling the desiccated nearshore basins and possibly even floating atop marine waters as the Mediterranean refilled from the west (Marzocchi et al., 2016; Orszag‐Sperber, 2006). Fossil fish deposits from this time period from the northern half of the Italian peninsula include a variety of euryhaline gobies (Carnevale, Dela Pierre, Natalicchio, & Landini, 2018; Carnevale, Landini, & Sarti, 2006), but they are mostly species of *Gobius* (family Gobiidae, *Gobius* lineage, Thacker & Roje, 2011) rather than sand gobies (family Gobionellidae, *Pomatoschistus* lineage). However, sand goby fossils are known from throughout the Middle Miocene of Croatia and Serbia (Schwarzhans et al., 2017). Freshwater sand gobies may have become isolated in the Paratethys and then experienced their crown diversification in the Adriatic and Aegean basins during the Lago Mare phase (*Knipowitschia* and *Orsinigobius*) or later in the Pliocene (*Ninnigobius*), remaining in marginal brackish and fresh waters after the Mediterranean refilled. Today, *Ninnigobius* and *Orsinigobius* are known only from drainages of the Adriatic, and *Economidichthys* only from the Ionian coast of Greece. Several *Knipowitschia* species are also endemic to that area (*K. montenegrina*,* K. panizzae*,* K. milleri*), others inhabit the Aegean coasts of Greece and Turkey (*K. thessala*,* K. byblisia*,* K. caunosi*,* K. mermere*), and the Black and Caspian seas (*K. longicaudata*,* K. iljini*,* K. bergi*, as well as the widespread *K. caucasica*). These distributions, combined with the inferred divergence dates and clade relationships, are consistent with release from the Paratethys and subsequent diversification of freshwater and brackish sand gobies at the Lago Mare phase of the MSC and afterward into the Pliocene and Pleistocene (Huyse et al., 2004; Miller, 1990; Thacker et al., 2019).

### 4.3 Evolutionary shape change and convergence among freshwater sand gobies

Sand gobies inhabiting fresh to brackish waters are not monophyletic, each genus is independently derived and they are interspersed phylogenetically with marine *Pomatoschistus* species, including a separate lineage for *P. quagga*, as shown in Figure 5. They also arose and then diversified at different times throughout the Miocene, Pliocene, and Pleistocene. Morphometrically, all of the freshwater species occupy a distinct region of morphospace, with the exception of *Ninnigobius*, the earliest‐diverging genus. *Ninnigobius* has a more generalized morphology than the other freshwater species (Figure 1); in particular, the relative enlargement of the head is less pronounced. CVA's and Procrustes ANOVAs of shape data (not phylogenetically corrected) show significant divergence among species in different habitats, but the distinction is not significant when corrected for phylogenetic relatedness. However, the pattern‐based tests of convergence among the freshwater species were highly significant, with convergence accounting for 40.2% of the variation. Unsurprisingly, variation in form among these species includes both phylogenetic and convergent components.

Convergence in morphology may be the result of a variety of evolutionary processes, including response to a common selective pressure, restriction by some developmental, genetic, or functional constraint, or even simple coincidence (Burns & Sidlauskas, 2019; Sanderson & Hufford, 1996). In the case of sand gobies, freshwater species have evolved similar overall shapes as they have diversified in different regions and at different times. What the species have in common is a change from a generally more expansive (marine) habitat into a restricted one, either nearshore brackish waters, rivers, streams, or tiny springs. It is likely that in these environments a smaller body size would be favored, for reasons including lower food requirements, ease of camouflage, and potentially more rapid growth and shorter generation time. In particular, if there is pressure for a more rapid maturation, which would be favored in a less stable habitat, it is possible that the small body size and proportional changes in the head and body shape are the result of heterochrony. A quickening of development in freshwater sand goby species could result in the overall convergent morphological changes documented here, even though the shape changes are not themselves the targets of selection. Freshwater sand gobies share reductive morphological changes in the head canals and scalation that are characteristic of subadult developmental stages (Economidis & Miller, 1990) and attain a generally smaller size. Lifespan data are scarce for these species, but *Ninnigobius canestrinii* and both *Economidichthys* species have lifespans of only one year, *Knipowitschia caucasica* may live for 1–2 years, and *Pomatoschistus microps* can live for 2–3 years (Miller, 1990, 2004). Overall, the sand goby genera exhibit a continuum of shorter lifespan, morphological reduction, and proportional enlargement of the head and shortening of the body that is correlated with increased freshwater preference, and consistent with a common heterochronic process of paedomorphosis.

## CONFLICT OF INTEREST

The authors declare no conflicts of interest.

## AUTHORS CONTRIBUTION

CT examined specimens and collected morphometric data. CG performed fieldwork and collected DNA data. CT performed analyses. Both authors wrote and edited the manuscript.

## Data Availability

Museum specimens: Specimens examined for this study are listed in the Appendix, 3A1. DNA sequences: GenBank accession numbers are listed in the Appendix, 4A2. DNA alignment and phylogeny, and morphometric data are available from Dryad https://doi.org/10.5061/dryad.c0qt85m.

## APPENDIX 1

**Table A1:** Museum catalog numbers for specimens examined in this study

Species	Catalog number	Number examined
*Economidichthys pygmaeus*	NMW33930	2
*Economidichthys pygmaeus*	NMW86068	6
*Economidichthys pygmaeus*	NMW86245	3
*Economidichthys pygmaeus*	BMNH1987	1
*Knipowitschia caucasica*	NMW91652	3
*Knipowitschia caucasica*	NMW91653	3
*Knipowitschia milleri*	NMW90127	1
*Knipowitschia milleri*	NMW90253	2
*Knipowitschia milleri*	NMW86065	1
*Knipowitschia milleri*	NMW86066	3
*Knipowitschia milleri*	NMW86067	2
*Knipowitschia panizzae*	NMW29805	5
*Knipowitschia panizzae*	NMW29806	4
*Knipowitschia panizzae*	NMW29808	3
*Knipowitschia panizzae*	NMW29810	1
*Ninnigobius canestrinii*	NMW29943	1
*Ninnigobius canestrinii*	NMW30372	6
*Ninnigobius canestrinii*	NMW30618	1
*Ninnigobius canestrinii*	NMW29956	2
*Orsinigobius punctatissimus*	NMW87514	6
*Pomatoschistus flavescens*	MNHN1995‐048	1
*Pomatoschistus flavescens*	MNHN1995‐050	1
*Pomatoschistus flavescens*	MNHN1995‐051	1
*Pomatoschistus flavescens*	MNHN1995‐053	1
*Pomatoschistus flavescens*	MNHN1995‐058	1
*Pomatoschistus lozanoi*	BMNH1951	1
*Pomatoschistus marmoratus*	NMW29363	2
*Pomatoschistus marmoratus*	NMW29364	2
*Pomatoschistus marmoratus*	NMW29953	3
*Pomatoschistus marmoratus*	NMW29956	2
*Pomatoschistus marmoratus*	NMW37513	1
*Pomatoschistus marmoratus*	NMW77868	1
*Pomatoschistus marmoratus*	NMW79810	1
*Pomatoschistus marmoratus*	NMW87354	1
*Pomatoschistus marmoratus*	NMW87356	3
*Pomatoschistus marmoratus*	NMW87360	1
*Pomatoschistus marmoratus*	NMW87361	3
*Pomatoschistus marmoratus*	NMW87362	4
*Pomatoschistus marmoratus*	NMW87363	1
*Pomatoschistus marmoratus*	BMNH2001	1
*Pomatoschistus microps*	NMW37505	1
*Pomatoschistus microps*	NMW37506	1
*Pomatoschistus microps*	NMW37507	1
*Pomatoschistus microps*	NMW37508	1
*Pomatoschistus microps*	NMW79819	2
*Pomatoschistus microps*	BMNH2001	1
*Pomatoschistus minutus*	NMW14375	7
*Pomatoschistus minutus*	NMW34370	4
*Pomatoschistus minutus*	BMNH2004	1
*Pomatoschistus norvegicus*	NMW98766	3
*Pomatoschistus pictus*	NMW28647	1
*Pomatoschistus pictus*	NMW28648	1
*Pomatoschistus pictus*	NMW28663	1
*Pomatoschistus pictus*	NMW28664	1
*Pomatoschistus pictus*	NMW28670	2
*Pomatoschistus pictus*	NMW28671	1
*Pomatoschistus pictus*	NMW28673	1
*Pomatoschistus pictus*	NMW28675	1
*Pomatoschistus pictus*	NMW37443	1
*Pomatoschistus pictus*	NMW37444	1
*Pomatoschistus pictus*	NMW37445	1
*Pomatoschistus pictus*	NMW81595	4
*Pomatoschistus pictus*	BMNH1988	1
*Pomatoschistus quagga*	NMW37657	3

**Table A2:** GenBank accession numbers for specimens analyzed in this study

KJ553311	*E. pygmaeus*
KJ553364	*E. pygmaeus*
KJ553482	*E. pygmaeus*
KJ553547	*E. pygmaeus*
KJ553595	*E. pygmaeus*
KJ553631	*E. pygmaeus*
KX673894	*E. pygmaeus*
KX673895	*E. pygmaeus*
KX673896	*E. pygmaeus*
KX673897	*E. pygmaeus*
KX673898	*E. pygmaeus*
KX673899	*E. pygmaeus*
KX673900	*E. pygmaeus*
KX673901	*E. pygmaeus*
KX673902	*E. pygmaeus*
KX673903	*E. pygmaeus*
KX673904	*E. pygmaeus*
KX673905	*E. pygmaeus*
KX673906	*E. pygmaeus*
KX673907	*E. pygmaeus*
KX673908	*E. pygmaeus*
KX673909	*E. pygmaeus*
KX673910	*E. pygmaeus*
KX673911	*E. pygmaeus*
KX673912	*E. pygmaeus*
KX673913	*E. pygmaeus*
KX673914	*E. pygmaeus*
KX673915	*E. pygmaeus*
KJ553316	*E. trichonis*
KJ553325	*E. trichonis*
KJ553425	*E. trichonis*
KJ553450	*E. trichonis*
KJ553457	*E. trichonis*
KX673893	*E. trichonis*
KJ553322	*K. byblisia*
KJ553368	*K. byblisia*
KJ553411	*K. byblisia*
KJ553412	*K. byblisia*
KJ553454	*K. byblisia*
KJ553539	*K. byblisia*
KJ553570	*K. byblisia*
KJ553602	*K. byblisia*
KJ553641	*K. byblisia*
HQ600736	*K. caucasica*
KJ553350	*K. caucasica*
KJ553351	*K. caucasica*
KJ553376	*K. caucasica*
KJ553407	*K. caucasica*
KJ553419	*K. caucasica*
KJ553420	*K. caucasica*
KJ553455	*K. caucasica*
KJ553557	*K. caucasica*
KJ553566	*K. caucasica*
KJ553584	*K. caucasica*
KJ553633	*K. caucasica*
KX673944	*K. caucasica*
KX673945	*K. caucasica*
KX673946	*K. caucasica*
KX673947	*K. caucasica*
KX673948	*K. caucasica*
KX673949	*K. caucasica*
KX673950	*K. caucasica*
KX673951	*K. caucasica*
KX673952	*K. caucasica*
KX673953	*K. caucasica*
KX673954	*K. caucasica*
KX673955	*K. caucasica*
KX673956	*K. caucasica*
KX673957	*K. caucasica*
KX673958	*K. caucasica*
KX673959	*K. caucasica*
KX673960	*K. caucasica*
KX673961	*K. caucasica*
KX673962	*K. caucasica*
KX673963	*K. caucasica*
KJ553390	*K. ephesi*
KJ553459	*K. ephesi*
KJ553609	*K. ephesi*
KJ553345	*K. mermere*
KJ553363	*K. mermere*
KJ553410	*K. mermere*
KJ553448	*K. mermere*
KJ553452	*K. mermere*
KJ553480	*K. mermere*
KJ553611	*K. mermere*
KJ553365	*K. milleri*
KJ553395	*K. milleri*
KJ553398	*K. milleri*
KJ553444	*K. milleri*
KJ553465	*K. milleri*
KJ553512	*K. milleri*
KJ553527	*K. milleri*
KJ553530	*K. milleri*
KJ553558	*K. milleri*
KJ553576	*K. milleri*
KJ553591	*K. milleri*
KJ553658	*K. milleri*
KX673916	*K. milleri*
KX673917	*K. milleri*
KX673918	*K. milleri*
KX673919	*K. milleri*
KX673920	*K. milleri*
KX673921	*K. milleri*
KX673922	*K. milleri*
KX673923	*K. milleri*
KX673924	*K. milleri*
KX673925	*K. milleri*
KX673926	*K. milleri*
KX673927	*K. milleri*
KX673928	*K. milleri*
KX673929	*K. milleri*
KX673930	*K. milleri*
KX673931	*K. milleri*
KX673932	*K. milleri*
KX673933	*K. milleri*
KX673934	*K. milleri*
KX673935	*K. milleri*
KX673936	*K. milleri*
KX673937	*K. milleri*
KX673938	*K. milleri*
KX673939	*K. milleri*
KJ553378	*K. montenegrina*
KJ553384	*K. montenegrina*
KJ553417	*K. montenegrina*
KJ553451	*K. montenegrina*
KJ553543	*K. montenegrina*
KJ553352	*K. mrakovcici*
KJ553370	*K. mrakovcici*
KJ553375	*K. mrakovcici*
KJ553514	*K. mrakovcici*
KJ553521	*K. mrakovcici*
KJ553537	*K. mrakovcici*
KJ553555	*K. mrakovcici*
KJ553594	*K. mrakovcici*
KJ552355	*K. panizzae*
KJ553310	*K. panizzae*
KJ553342	*K. panizzae*
KJ553355	*K. panizzae*
KJ553377	*K. panizzae*
KJ553400	*K. panizzae*
KJ553423	*K. panizzae*
KJ553424	*K. panizzae*
KJ553437	*K. panizzae*
KJ553468	*K. panizzae*
KJ553469	*K. panizzae*
KJ553491	*K. panizzae*
KJ553511	*K. panizzae*
KJ553579	*K. panizzae*
KJ553613	*K. panizzae*
KJ553643	*K. panizzae*
KJ553652	*K. panizzae*
KX673964	*K. panizzae*
KX673965	*K. panizzae*
KX673966	*K. panizzae*
KX673967	*K. panizzae*
KJ553466	*K. radovici*
KJ553481	*K. radovici*
KJ553552	*K. radovici*
KJ553599	*K. radovici*
KJ553636	*K. radovici*
KJ553656	*K. radovici*
KJ553439	*K. thessala*
KJ553464	*K. thessala*
KJ553503	*K. thessala*
KX673940	*K. thessala*
KX673941	*K. thessala*
KX673942	*K. thessala*
KX673943	*K. thessala*
KJ553809	*N. canestrinii*
KJ554046	*N. canestrinii*
KJ554069	*N. canestrinii*
KX673968	*N. canestrinii*
KX673969	*N. canestrinii*
KX673970	*N. canestrinii*
KX673971	*N. canestrinii*
KJ553666	*N. montenegrensis*
KJ553703	*N. montenegrensis*
KJ553759	*N. montenegrensis*
KJ554044	*N. montenegrensis*
KJ554070	*N. montenegrensis*
KJ553751	*O. croaticus*
KJ553780	*O. croaticus*
KJ553811	*O. croaticus*
KJ553834	*O. croaticus*
KJ553854	*O. croaticus*
KJ554032	*O. croaticus*
KJ554053	*O. croaticus*
KJ553912	*O. punctatissimus*
KJ553993	*O. punctatissimus*
KJ128503	*P. flavescens*
KJ128504	*P. flavescens*
KM077830	*P. flavescens*
KM077831	*P. flavescens*
KM077832	*P. flavescens*
KM077833	*P. flavescens*
KM077834	*P. flavescens*
KM077835	*P. flavescens*
JQ775029	*P. lozanoi*
JQ775030	*P. lozanoi*
JQ775031	*P. lozanoi*
JQ775032	*P. lozanoi*
JQ775033	*P. lozanoi*
KM077847	*P. lozanoi*
KM077848	*P. lozanoi*
KM077849	*P. lozanoi*
KJ554258	*P. marmoratus*
KJ554272	*P. marmoratus*
KJ554281	*P. marmoratus*
KJ554294	*P. marmoratus*
KJ554300	*P. marmoratus*
KJ554461	*P. marmoratus*
KJ709583	*P. marmoratus*
KJ709584	*P. marmoratus*
KX673972	*P. marmoratus*
KX673973	*P. marmoratus*
KX673974	*P. marmoratus*
KX673975	*P. marmoratus*
KJ128585	*P. microps*
KJ128586	*P. microps*
KJ554119	*P. microps*
KJ554147	*P. microps*
KJ554336	*P. microps*
KJ554517	*P. microps*
KJ768285	*P. microps*
KJ768286	*P. microps*
KJ768287	*P. microps*
KM077850	*P. microps*
KM077851	*P. microps*
KM077852	*P. microps*
KM077853	*P. microps*
KM077854	*P. microps*
KM077855	*P. microps*
KM077856	*P. microps*
KJ128587	*P. minutus*
KJ128588	*P. minutus*
KM077857	*P. minutus*
KM077858	*P. minutus*
KM077859	*P. minutus*
KM077860	*P. minutus*
KM077861	*P. minutus*
KM077862	*P. minutus*
KM077863	*P. minutus*
KM077864	*P. minutus*
KM077865	*P. minutus*
KM077866	*P. minutus*
KJ128589	*P. norvegicus*
KJ128590	*P. norvegicus*
KM077867	*P. norvegicus*
KM077868	*P. norvegicus*
KM077869	*P. norvegicus*
KM077870	*P. norvegicus*
KM077871	*P. norvegicus*
KM077872	*P. norvegicus*
KM077873	*P. norvegicus*
KM077874	*P. norvegicus*
KJ128591	*P. pictus*
KM077875	*P. pictus*
KM077876	*P. pictus*
KM077877	*P. pictus*
KM077878	*P. pictus*
MK302484	*P. quagga*
MK302485	*P. quagga*
MK302486	*P. quagga*
MK302487	*P. quagga*
MK302488	*P. quagga*
FJ751920	*P. tortonesi*
FJ751921	*P. tortonesi*
FJ751922	*P. tortonesi*
KJ709585	*P. tortonesi*
KJ709586	*P. tortonesi*
EU444698	*Zosterisessor ophiocephalus*
KM538356	*Gobius niger*
KM077839	*Gobius niger*
KM887159	*Mugilogobius adeia*
KM887169	*Mugilogobius hitam*
KM887161	*Mugilogobius latifrons*
